# Perioperative fluid balance and postoperative changes in serum creatinine in patients admitted to critical care after elective major surgery

**DOI:** 10.1186/cc13335

**Published:** 2014-03-17

**Authors:** R Rajendram, JR Prowle

**Affiliations:** 1 Barts Health NHS Trust, London, UK

## Introduction

The rationale for perioperative fluid therapy has been to preserve organ perfusion. However, conservative postoperative fluid balance (FB) is now encouraged to avoid the effects of iatrogenic fluid overload. In addition, fluid expansion has been described as a confounder of acute kidney injury (AKI) diagnosis by dilution serum creatinine (SCr).

## Methods

We conducted a prospective audit of FB and renal function in patients undergoing major elective surgery admitted to critical care over a 28-day period. Fluid overload was defined as: (positive FB) / (weight × 0.6) × 100%.

## Results

**Figure 1:**
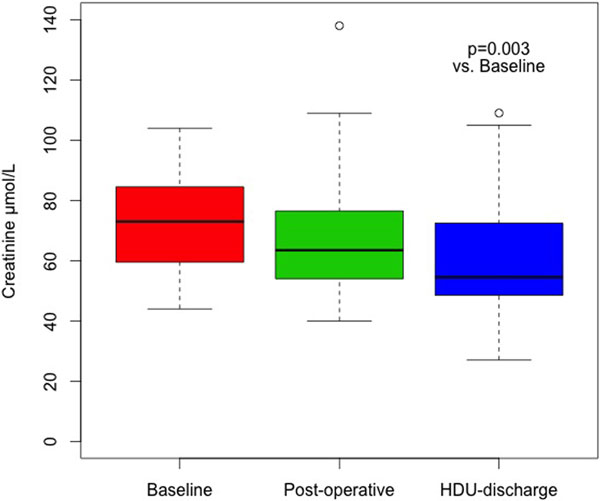
**Serum creatinine changes after major surgery**.

Thirty-two patients (56% female, median age 64) were studied over a median of 3 critical care days (range 1 to 7). Total FB was +3.9 l at discharge; however, 75% of this occurred intraoperatively so that positive FB in critical care was only +390 ml/day. Patients had median 9% fluid overload at discharge. Two patients had transient AKI stage 1. Overall SCr decreased significantly from preoperatively to discharge (*P *= 0.003), median 73 to 55 μmol/l, with decreases occurring both postoperatively and during critical care stay (Figure 1).

## Conclusion

We achieved near-neutral fluid balances after admission, but did not resolve intraoperative fluid accumulation. SCr fell significantly, even after there was no further net fluid accumulation. This suggests a decrease in creatinine generation after major surgery, rather than fluid expansion, may largely account for sCr decreases in these patients.

